# Organophosphate Insecticide Metabolites in Prenatal and Childhood Urine Samples and Intelligence Scores at 6 Years of Age: Results from the Mother–Child PELAGIE Cohort (France)

**DOI:** 10.1289/ehp.1409472

**Published:** 2015-09-22

**Authors:** Chloé Cartier, Charline Warembourg, Gaïd Le Maner-Idrissi, Agnès Lacroix, Florence Rouget, Christine Monfort, Gwendolina Limon, Gaël Durand, Dave Saint-Amour, Sylvaine Cordier, Cécile Chevrier

**Affiliations:** 1 INSERM U1085-IRSET, Université Rennes I, Rennes, France; 2 Département de psychologie, Université du Québec à Montréal, Montréal, Québec, Canada; 3 Centre de Recherches en Psychologie, Cognition et communication (CRPCC EA 1285), Université Rennes 2, Rennes, France; 4 ”Bien Naître en Ille et Vilaine” network, Rennes, France; 5 LABOCEA Laboratoire, Plouzané, France; 6 Centre de recherche et département d’ophtalmologie, CHU Sainte-Justine, Montréal, Québec, Canada

## Abstract

**Background:**

Several studies suggest that exposure to organophosphate insecticides (OP) during pregnancy impairs neurodevelopment in children.

**Objectives:**

We evaluated associations between biomarkers of prenatal and postnatal OP exposure and cognitive function of 6-year-olds in a French longitudinal birth cohort.

**Methods:**

In 2002–2006, the PELAGIE mother–child cohort enrolled pregnant women from Brittany. For a random subcohort, we measured nonspecific dialkylphosphate metabolites (DAP) of OP in one maternal urine sample, collected before 19 weeks’ gestation, and in one urine sample collected from their 6-year-old children. Six subtests of the Wechsler Intelligence Scale for Children, 4th edition (WISC-IV) were administered when the children were 6 years of age to evaluate cognitive function (n = 231). Linear regression models controlling for factors including maternal intelligence and the Home Observation for Measurement of the Environment score were used.

**Results:**

WISC-IV scores were not significantly associated with prenatal or childhood total DAP metabolites. WISC verbal comprehension score was significantly higher in association with the highest maternal urinary concentrations of diethylphosphate (DE) metabolites (5.5; 95% CI: 0.8, 10.3 for > 13.2 nmol/L vs. < LOQ), whereas WISC working memory score was significantly lower in association with the highest urinary concentrations of DE metabolites at age 6 years (–3.6; 95% CI: –7.8, –0.6 for > 11.1 nmol/L vs. < LOD).

**Conclusion:**

We found no evidence that prenatal OP exposure adversely affected cognitive function in 6-year-olds, perhaps because of the population’s socioeconomic status, which was higher than in previous studies, though other causal and noncausal explanations are also possible. The negative association between WISC score and concurrent DE urinary concentrations requires replication by longitudinal studies investigating childhood OP exposure.

**Citation:**

Cartier C, Warembourg C, Le Maner-Idrissi G, Lacroix A, Rouget F, Monfort C, Limon G, Durand G, Saint-Amour D, Cordier S, Chevrier C. 2016. Organophosphate insecticide metabolites in prenatal and childhood urine samples and intelligence scores at 6 years of age: results from the mother–child PELAGIE cohort (France). Environ Health Perspect 124:674–680; http://dx.doi.org/10.1289/ehp.1409472

## Introduction

Organophosphate insecticides (OPs) are used on conventional agricultural crops and for residential and veterinary purposes. In France, these chemicals have been found in dietary products (Nougadère et al. 2012), outdoor air (Air Breizh 2012), and indoor environments; residues have been detected in house dust (Bouvier et al. 2006b) and indoor air (Bouvier et al. 2006b) and measured on the hands of both children and adults (Bouvier et al. 2006a). Accordingly, exposure to OPs is thought to occur via dietary intake (Lu et al. 2008), direct contact from domestic use, and proximity to spraying areas. Numerous biological monitoring studies show that OP exposure is widespread among children and pregnant women (Bradman et al. 2013; Castorina et al. 2010). The finding of OPs in amniotic fluid and meconium from newborns (Berton et al. 2014; Bradman et al. 2003) suggests that fetuses too are exposed to these chemicals. After birth, babies and toddlers can be exposed to OPs via breastfeeding (Weldon et al. 2011) and repeated hand-to-mouth behavior.

OP insecticides are well-known neurotoxicants (Costa 2006). At high doses, these chemicals inhibit acetyl cholinesterase and thus result in a buildup of acetylcholine in synaptic junctions. At doses too low to inhibit acetyl cholinesterase, studies in animals indicate that OPs alter gene expression, reduce neuronal survival and differentiation, and influence non-cholinergic biochemical pathways (Costa 2006; Slotkin et al. 2006). The association of chronic low doses of OPs with child development is currently under active investigation. Because the brain is more vulnerable to neurotoxic insult during maturational and developmental processes (Rice and Barone 2000), perinatal and childhood exposures may constitute a window of higher susceptibility to neurotoxicity.

Several epidemiological studies report associations between perinatal OP exposure and adverse neurodevelopmental outcomes among children (González-Alzaga et al. 2014). Using questionnaire-based assessment of occupational exposure, studies have reported that children exposed *in utero* to OPs in their mothers’ workplaces appear to have impaired visual memory (Handal et al. 2008) and altered motor function (Grandjean et al. 2006; Handal et al. 2008; Harari et al. 2010). Longitudinal birth cohort studies using maternal exposure biomarkers report that prenatal OP exposure has been associated with poorer reflexes in newborns (Engel et al. 2007; Young et al. 2005), mental and psychomotor developmental delays in children (Engel et al. 2011; Eskenazi et al. 2007; Rauh et al. 2006), lower social skills (Furlong et al. 2014), decreased intellectual functioning (Bouchard et al. 2011; Engel et al. 2011; Rauh et al. 2011), and higher rates of children who screen positive for symptoms of attention deficit/hyperactivity disorder (Marks et al. 2010). These longitudinal cohort studies were all conducted in North America, and despite the variety of the neurodevelopmental tests used in these studies they have reached the same conclusions: Prenatal OP exposure may be associated with developmental neurotoxicity.

The aim of this study was to investigate the association between prenatal and postnatal OP exposure assessed with urinary biomarkers and cognitive neurodevelopment in 6-year-old children from a European longitudinal study.

## Methods

*Population and study area.* The PELAGIE cohort, which included 3,421 pregnant women enrolled between May 2002 and February 2006 in Brittany (France), was established to assess the potential impact of environmental and occupational exposure to chemicals on pregnancy and child development. Brittany is an important French agricultural region with 65% of its area devoted to agriculture in 2006, particularly maize, grains, and vegetables (Agreste Bretagne 2007). Pregnant women were enrolled before 19 weeks’ gestation from the general population by obstetricians, gynecologists, or ultrasonographers at early visits for prenatal care. They were asked to complete a questionnaire including their home address; social, demographic, occupational, and medical characteristics; dietary habits (fish, fruit and vegetable, dairy products, eggs, meat, offal, and bread); and lifestyle. Most reported household use of insect control products during the pregnancy (Petit et al. 2012).

A subcohort of 601 women (18% of the total cohort) was randomly selected for biological assays from the cohort members who gave birth to a live-born singleton (Chevrier et al. 2011). For the present analysis, we excluded subcohort members if the child’s gestational age at birth was < 35 weeks; if the child underwent neonatal hospitalization or resuscitation, or had hypoglycemia or a 5-min Apgar score < 7 at delivery; if the child was diagnosed with genetic anomalies; or the mother or child died after birth; or the child was > 6 years of age at the time of the study (i.e., was born before September 2003). This left 477 eligible families. Among them, we were able to contact 373 families by telephone and invite them to participate in the neuropsychological examination (before the child reached 6 years, 3 months of age). Fifteen children had already undergone neuropsychological tests, mainly for speech therapy, and were thus excluded. Another 115 families refused to participate, mostly because of lack of time. The participation rate was 65% (243/373 families we could contact). Among these participants, 231 of the mothers had prenatal urinary samples available. At the time of the neuropsychological examination, all mothers completed additional questionnaires about their 6-year-old child’s environment and health. The study subjects gave written informed consent before participating in this follow-up study, and the appropriate French ethics committees approved all study procedures.

*Measurements of OP exposure.* At enrollment, pregnant women were asked to collect, at home, the first-morning-void urine sample into 10-mL vials (containing nitric acid to prevent bacterial multiplication). They returned the urine sample to the research laboratory by local mail, in a self-addressed prestamped package. At reception, samples were frozen at –20°C until shipment to the analysis laboratory. Children at age 6 years were asked to provide the first-morning-void urine sample, which was provided to the psychologist at the time of the home visit. First-morning-void urine samples were chosen because they are more concentrated than urine samples collected at other times (Barr et al. 2005).

One urine sample from each mother and one from each child were analyzed for six nonspecific dialkylphosphate (DAP) metabolites of numerous OP insecticides [diethylphosphate (DEP), diethylthiophosphate (DETP), diethyldithiophosphate (DEDTP), dimethylphosphate (DMP), dimethylthiophosphate (DMTP), and dimethyldithiophosphate (DMDTP)]. The chemical analyses were performed by the LABOCEA Institute (Plouzané, France) with solid-phase extraction (SPE) and liquid chromatography-electrospray ionization tandem mass spectrometry (LC/MS-MS). The mothers’ samples (10 mL) were analyzed in 2008, and the children’s (1 mL) in 2013 with improved equipment. For a detailed description of the urine assay methods, including quality-control/quality assurance procedures and equipment, see Supplemental Material, “Chemical analyses.”

The limits of quantification (LOQ) for the chemical analyses of maternal urine samples were 1.25, 1.7, 0.02, 0.2, 1, and 0.45 μg/L for, respectively, DEP, DETP, DEDTP, DMP, DMTP, and DMDTP, and the coefficients of variation at LOQ respectively 19, 19, 20, 17, 19, and 20%. For the children’s samples, values between the limit of detection (LOD) and the LOQ were available. The LODs were 0.2, 0.1, 0.005, 0.06, 0.32, and 0.13 μg/L, and the coefficients of variation 20, 17, 18, 16, 13, and 17% for, respectively, DEP, DETP, DEDTP, DMP, DMTP, and DMDTP. For the sake of clarity, hereafter we use LOD to refer to LOQ for maternal urinary samples.

Metabolite concentrations were converted from micrograms per liter to their molar concentrations (nanomoles per liter). Concentrations were summed to obtain overall concentrations of diethylphosphate metabolites (DE; sum of DEP, DETP, and DEDTP), dimethylphosphate metabolites (DM; sum of DMP, DMTP, and DMDTP), and all six nonspecific dialkylphosphate metabolites.

*Neuropsychological assessment and questionnaires.* The Wechsler Intelligence Scale for Children, 4th edition, French version (WISC-IV), was used to assess children’s cognitive functions at the age of 6 years (Wechsler 2003). Assessments were conducted at home by one of the two trained psychologists, blinded to the child’s prenatal and current OP exposure. Six WISC-IV subtests were administered to assess cognitive abilities: *a*) Similarities, *b*) Vocabulary, *c*) Comprehension, *d*) Digit Span, *e*) Letter–Number Sequencing, and *f*) Block Design. Two scores were calculated with the age-standardized WISC-IV norms: *a*) working memory score (Digit Span and Letter–Number Sequencing subtests), and *b*) verbal comprehension score (Similarities, Vocabulary, and Comprehension subtests). A few children had incomplete testing because they expressed fatigue. For the WISC-IV working memory score, the Letter–Number Sequencing subtest was missing for 8 children. For the WISC-IV verbal comprehension score, missing values were from missing Comprehension subtest (*n* = 2) or from two missing subtests (Comprehension and Vocabulary, *n* = 1). The scores were highly correlated with their respective subtests (Pearson’s rho ranging from 0.74 to 0.89, all *p* < 0.0001) allowing us to predict the missing scores from their available subtests with linear regression model. The second psychologist assessed simultaneously the verbal IQ of the mother at the home, with the Wechsler Adult Intelligence Scale, 3rd edition (WAIS-III) (Wechsler 1997), as well as the family environment, with the Home Observation for Measurement of the Environment inventory (HOME) (Caldwell and Bradley 1984).

*Potential covariables.* The following potential confounding variables were chosen based on prior knowledge of factors influencing neurodevelopment: *a*) maternal characteristics at inclusion (< 19 weeks of gestation): mothers’ age, education (university level or lower), tobacco and alcohol use during pregnancy (yes or no), usual consumption of fish (≥ 2 per week or less), usual fruit and vegetable consumption (≥ 3 per day or less), urinary creatinine levels (continuous variable), season of urine collection (spring, summer, autumn, or winter), and parity (0 or ≥ 1); *b*) family characteristics at the 6-year follow-up: marital status (living alone or not), maternal verbal IQ (continuous variable), HOME score (continuous variable), and number of children before the index child (continuous variable); *c*) children’s characteristics: breastfeeding duration (no, ≤ 16 weeks, or > 16 weeks), school (preschool or elementary school), and urinary creatinine levels; and *d*) testing characteristics: psychologist testing the child and any disturbances during the testing (yes or no). Missing values for categorical covariates were imputed using the most common category (breastfeeding duration: *n* = 1, > 16 weeks; school: *n* = 1, primary school; mother’s marital status: *n* = 8, married/cohabitating), and children with missing data urine creatinine (*n* = 3) were assumed to have the median value for all children (920 mg/L).

*Statistical analysis.* We examined the association between urinary concentrations of OP metabolites and WISC scores with two approaches. In the first, with no prior assumption about the shape of these relations, we categorized the urinary concentrations into three groups for DE: *a*) values < LOD, *b*) values > LOD and ≤ median, and *c*) values > LOD and > median; we categorized the values for DAP and DM in tertiles with equal-sized groups; and we conducted linear regression models. For the second approach, we conducted restricted cubic spline regressions of continuous natural log–transformed concentrations to explore the possible dose–response relation and exploit the finer resolution of our exposure data. For these models, values < LOD were randomly imputed from a lognormal-probability distribution that was derived using a maximum-likelihood estimation method [Jin et al. 2011; with the PROC LIFEREG with SAS software (SAS Institute Inc.)]. Spline model estimates with 95% confidence intervals (CIs) were graphed (SAS macro %RCS_Reg; Desquilbet and Mariotti 2010), and the difference in the fit of the spline models and linear models was tested. If the nonlinear model did not significantly improve the model fit (*p* > 0.05), we also report coefficients from linear models of the log-transformed and imputed exposures. For both approaches, separate regression models were performed to estimate associations with DAP, DM, and DE metabolites, respectively. Models for each set of metabolites included both prenatal and childhood exposures (either categorical or splined) in the same model.

For the linear regression model, covariates were selected if they were potential risk factors associated with both prenatal urinary concentrations and WISC scores at *p* ≤ 0.15 (i.e., total HOME score, breastfeeding duration, and child school), or if they were risk factors highly associated with WISC scores (*p* ≤ 0.005; i.e., maternal verbal IQ and education level, and the psychologist). Additional covariates and their potential confounding effects were then considered one by one in each multivariate model; they were retained if they modified the values of the coefficients of the prenatal exposure variable by ≥ 10%. To investigate the association between OP postnatal exposure and WISC scores, the children’s OP metabolite urinary concentrations were forced in the final regression models, as were the mothers’ and children’s creatinine levels. For spline models, adjustments were similar to those used in the linear regression models.

Because associations have been reported between prenatal DAP urinary concentrations and birth weight (Rauch et al. 2012; Whyatt et al. 2004), the latter may lie on the causal pathway between exposure and cognitive scores. Thus, in additional analyses, the final regression models were rerun with birth weight as a covariate. Additional sensitivity analyses were performed including only participants with complete data (for exposure variables, covariates, and WISC-IV subtest scores, *n* = 216).

Tests were two-sided and conducted at the α = 0.05 level. All analyses were performed with SAS software (SAS/STAT version 9.3).

## Results

Table 1 summarizes the descriptive characteristics of the 231 families included in this study. The mothers’ average age at enrollment was 30.3 years. Most had attended university (68.0%) and lived with the child’s father (94.2%). At inclusion, 23.4% reported smoking and 13% drinking alcoholic beverages at least once a week at the beginning of pregnancy. Girls and boys were equally represented in the sample, and 65% of the children had been breastfed.

**Table 1:** Descriptive characteristics of the participants (*n *= 231; PELAGIE cohort, France).

Characteristics of mothers and families	*n* (%)	Mean ± SD	Range
At inclusion during pregnancy (< 19 weeks gestation)
Mothers’ age (years)	231	30.3 ± 4.1	21.9–44
Maternal educational level
High school or less	74 (32.0)
University level	157 (68.0)
Smoking (% yes)	231 (23.4)
Alcohol use (% yes)^*a*^	231 (13.0)
Fish intake (% ≥ 2 per week)	231 (29.4)
Fruit and vegetable consumption (% ≥ 3 per day)	231 (24.2)
Urinary creatinine levels (mg/L)	231	1,080 ± 509	235–3,511
Season of urine collection
Spring**	72 (31.2)
Summer**	58 (25.1)
Autumn**	48 (20.8)
Winter**	53 (22.9)
Parity
0	98 (42.4)
≥ 1**	133 (57.6)
At the 6-year-old follow-up
Marital status (% single)^*c*^	231 (5.8)
Mothers’ IQ^*b*^	231	93.4 ± 11.4	60–127
HOME score	231	46.1 ± 4.3	27–46.1
Number of children in the family at the 6-year follow-up	231	2.4 ± 0.7	1–6
Children’s characteristics
Sex (% male)	231 (50.6)
Birth weight (g)	231	3,401 ± 436	2,340–4,660
Breastfeeding duration^*c*^
No breastfeeding**	80 (34.6)
≤ 16 weeks**	70 (30.3)
> 16 weeks**	81 (35.1)
School at 6 years of age (% elementary)^*c*^	231 (27.7)
Testing characteristics
Child psychologist (% psychologist 1)	231 (50.2)
Disturbance during test (% yes)	231 (8.2)
^***a***^Drinking an alcoholic beverage at least once a week or more during pregnancy. ^***b***^Measured with the Wechsler Adult Intelligence Scale, 4th ed. ^***c***^Missing values were imputed for breastfeeding duration (*n *= 1, > 16 weeks), school (*n *= 1, primary school), and mother’s marital status (*n *= 8, married/cohabitating).			

Of the 231 mothers included in the present study, 68% had attended university compared to 53.9% among the 246 mothers of children who were eligible for the 6-year neuropsychological follow-up but were not included (see Supplemental Material, Table S1). Maternal age at birth, smoking during pregnancy, and birth weight were not significantly different between the two groups, but fruit and vegetable consumption during pregnancy was significantly higher among women included in the present analysis (24.2% vs. 9.8% reporting consumption ≥ 3 times per day).

Among the 231 participating children, the mean (± SD) WISC-IV working memory score was 107 ± 14 and the mean WISC-IV verbal comprehension score was 107 ± 16) (Table 2). Higher maternal education level, maternal WAIS-III verbal IQ score, and HOME score were all significantly associated with higher WISC-IV scores (see Supplemental Material, Table S2). A higher WISC-IV working memory score was observed with elementary school (vs. preschool), higher fruit and vegetable consumption during pregnancy, and with mothers who did not smoke during pregnancy. WISC-IV memory score was also related to the psychologist testing the child. A higher WISC-IV verbal comprehension score was associated with breastfeeding duration, birth weight, fish intake, fruit and vegetable consumption, and no disturbance during testing.

**Table 2:** Age-standardized WISC-IV scores of 6-year-old children (*n *= 231; PELAGIE cohort, France).

WISC-IV index and subtest	Mean ± SD	p25	p50	p75	Minimum–maximum
Working memory index	107.3 ± 14.1	97	106	118	62–140
Digit Span	11.3 ± 2.8	9	12	14	3–19
Letter–Number Sequencing^*a*^	11.3 ± 2.4	9	11	13	7–18
Verbal comprehension index	107.0 ± 16.0	98	108	118	72–155
Similarities	11.9 ± 3.3	10	12	14	6–19
Vocabulary^*b*^	9.7 ± 3.5	8	10	12	1–19
Comprehension^*c*^	11.9 ± 3.1	10	12	14	3–19
p, percentile. ^***a***^Eight missing values. ^***b***^One missing value. ^***c***^Three missing values.					

As shown in Table 3, in the prenatal maternal urine samples, DAP, DM, and DE metabolites were quantified in 91.3%, 89.6%, and 49.8%, respectively. Median urinary concentrations were 43.9 nmol/L for DAP and 34.3 nmol/L for DM. In the children’s urine samples, DAP, DM, and DE metabolites were detected in 79.2%, 60.6%, and 52.8%, respectively. Median urinary concentrations were 11.3 nmol/L for DAP, 3 nmol/L for DM, and 0.8 nmol/L for DE (Table 3). Mothers’ OP urinary concentrations were not statistically significantly correlated with those of their children (in log-scale; DAP: *r*_Pearson_ = 0.02, *p* = 0.81; DM: *r*_Pearson_ = –0.04, *p* = 0.66; DE: *r*_Pearson_ = 0.06, *p* = 0.64).

**Table 3:** Quantification frequency and median molar concentrations of nonspecific OP metabolites in urine samples among all values (*n *= 231; PELAGIE cohort, France).

OP metabolites	% ≥ LOQ or LOD^*a*^	p10	p25	p50	p75	p90
Maternal urine samples
Dialkylphosphates	91.3	2.3	14.5	43.9	85.6	151.2
Dimethylphosphates	89.6	< LOQ	10.5	34.3	71.9	115.5
Diethylphosphates	49.8	< LOQ	< LOQ	< LOQ	13.2	36.2
Children’s urine samples (at age 6 years)
Dialkylphosphates	79.2	< LOD	1.8	11.3	34.1	120.6
Dimethylphosphates	60.6	< LOD	< LOD	3.0	19.9	65.0
Diethylphosphates	52.8	< LOD	< LOD	0.8	12.1	28.9
p, percentile. % ≥ LOQ (for maternal urine samples): proportion of urinary samples in the study population with a quantified value for the metabolites of interest, % ≥ LOD (for children’s urine samples): proportion of urinary samples in the study population with a detected value for the metabolites of interest. ^***a***^LOQ were 1.25, 1.7, 0.02, 0.2, 1, and 0.45 μg/L for DEP, DETP, DEDTP, DMP, DMTP, and DMDTP, respectively; LOD were 0.2, 0.1, 0.005, 0.06, 0.32, and 0.13 μg/L for DEP, DETP, DEDTP, DMP, DMTP, and DMDTP, respectively.						

Final regression models for WISC-IV working memory scores are presented in Table 4. After adjustment for the covariates, prenatal DAP, DM, and DE urinary levels were not statistically significantly associated with WISC-IV working memory scores measured at age 6 years.

**Table 4:** Associations between OP urinary metabolites and WISC-IV working memory scores (*n *= 231; PELAGIE cohort, France).

OP metabolites (nmol/L)	*n*	β (95% CI)
Pregnancy urinary samples
DAP^*a*^
< 22.2	77	Reference
22.2–68.8	77	–1.5 (–5.9, 2.9)
> 68.8	77	–0.6 (–5.0, 3.9)
Log-concentration	231	–0.55 (–1.44, 0.34)
DM^*b*^
< 15.5	77	Reference
15.5–59.9	77	–1.3 (–5.7, 3.1)
> 59.9	77	–0.6 (–5.1, 4.0)
Log-concentration	231	–0.64 (–1.81, 0.53)
DE^*c*^
< LOQ	116	Reference
> LOQ–13.2	58	–0.7 (–5.0, 3.6)
> 13.2	57	2.1 (–2.3, 6.4)
Log-concentration	231	0.13 (–0.29, 0.55)
6-year urinary samples
DAP^*a*^
< 3.95	77	Reference
3.95–25	76	–0.6 (–5.0, 3.7)
> 25	78	–2.6 (–6.9, 1.8)
Log-concentration	231	–0.23 (–1.03, 0.57)
DM^*b*^
< LOD	91	Reference
> LOD–13	70	–0.5 (–4.8, 3.8)
> 13	70	0.2 (–4.1, 4.5)
Log-concentration	231	0.07 (–0.51, 0.64)
DE^*c*^
< LOD	109	Reference
> LOD–11.1	61	–1.5 (–5.7, 2.8)
> 11.1	61	–3.6 (–7.8, –0.6)
Log-concentration	231	–0.33 (–0.76, 0.10)
Abbreviations: DAP, dialkyphosphate; DM, dimethylphosphate; DE, diethylphosphate. Urinary concentrations during pregnancy and during childhood were included simultaneously in the models. Models of natural log–transformed exposure concentrations were run after confirming *p* > 0.05 for departures from linearity. All models were adjusted for HOME score, breastfeeding duration, mothers’ IQ, school, maternal education level, psychologist testing the child, creatinine levels of mother and child, parity, and season of urine collection. ^***a***^DAP models were also adjusted for maternal alcohol use at inclusion, and disturbances during testing. ^***b***^DM models were also adjusted for maternal alcohol use at inclusion, disturbances during testing, marital status, maternal fruit and vegetable consumption, maternal fish intake, and child’s sex. ^***c***^DE models were also adjusted for marital status, maternal fish intake, and child’s sex.		

Simultaneously, no significant associations were observed between DAP or DM measured at 6 years of age and the WISC-IV working memory score. WISC-IV working memory scores were significantly lower in association with the highest category of DE in children’s urine samples (–3.6; 95% CI: –7.8, –0.6 for urinary concentrations > 11.1 nmol/L vs. < LOD).

Estimates from models that included only prenatal and childhood exposures and corresponding creatinine concentrations were generally consistent with estimates from the fully adjusted models, with no consistent evidence of associations between exposures and working memory scores (see Supplemental Material, Table S3). The association with high DE concentrations at age 6 years was negative but not significant (–2.1; 95% CI: –7.1, 1.9 for urinary concentrations > 11.1 nmol/L vs. < LOD).

In general, dose–response curves generated from spline models of log-transformed exposures were consistent with estimates from models of the exposures as categorical variables, without clear evidence of strong or consistent associations (see Supplemental Material, Figure S1). Statistical tests comparing the spline models to linear models of the log-transformed exposures did not indicate significant departures from linearity for any of the exposures (*p*-values ranging from 0.20 to 0.97). None of the coefficients for log-scale DAP concentrations in the linear models were statistically significant (for the DE concentration at age 6 years, β = –0.33; 95% CI: –0.76, 0.10, *p* = 0.13; Table 4).

Table 5 presents the final regression models for WISC-IV verbal comprehension scores. After adjustment, maternal DAP and DM urinary concentrations were not significantly associated with their children’s verbal comprehension scores. The highest maternal urinary concentrations of DE metabolites were associated with a better WISC-IV verbal comprehension score (5.5; 95% CI: 0.8, 10.3 for > 13.2 nmol/L vs. < LOQ).

**Table 5:** Associations between OP urinary metabolites and WISC-IV verbal comprehension scores (*n *= 231; PELAGIE cohort, France).

OP metabolites (nmol/L)	*n*	β (95% CI)
Pregnancy urinary samples
DAP^*a*^
< 22.2	77	Reference
22.2–68.8	77	3.4 (–1.4, 8.1)
> 68.8	77	3.0 (–1.8, 7.8)
Log-concentration	231	0.32 (–0.63, 1.28)
DM^*b*^
< 15.5	77	Reference
15.5–59.9	77	0.4 (–4.5, 5.3)
> 59.9	77	1.1 (–3.9, 6.2)
Log-concentration	231	0.37 (–0.93, 1.66)
DE^*c*^
< LOQ	116	Reference
> LOQ–13.2	58	–0.7 (–5.4, 4.0)
> 13.2	57	5.5 (0.8, 10.3)
Log-concentration	231	0.33 (–0.12, 0.79)
6-year urinary samples
DAP^*a*^
< 3.95	77	Reference
3.95–25	76	1.8 (–3.0, 6.5)
> 25	78	–1.3 (–6.1, 3.5)
Log-concentration	231	–0.45 (–1.32, 0.42)
DM^*b*^
< LOD	91	Ref
> LOD–13	70	–0.1 (–4.9, 4.6)
> 13	70	–2.6 (–7.3, 2.1)
Log-concentration	231	–0.33 (–0.96, 0.31)
DE^*c*^
< LOD	109	Reference
> LOD–11.1	61	–0.4 (–5.1, 4.2)
> 11.1	61	–0.9 (–5.5, 3.7)
Log-concentration	231	–0.03 (–0.51, 0.44)
Abbreviations: DAP, dialkyphosphate; DM, dimethylphosphate; DE, diethylphosphate. Urinary concentrations during pregnancy and during childhood were included simultaneously in the models. Models of natural log–transformed exposure concentrations were run after confirming *p* > 0.05 for departures from linearity. All models were adjusted for HOME score, breastfeeding duration, mothers’ IQ, school, maternal education level, psychologist testing the child, and creatinine levels of mother and child. ^***a***^DAP models were also adjusted for disturbances during testing. ^***b***^DM models were also adjusted for disturbances during testing, parity, season of urine collection, maternal fruit and vegetable consumption, and child’s sex. ^***c***^DE models were also adjusted for maternal fish intake.		

In the same regression models, no statistically significant associations were observed between the children’s urinary concentrations of DAP, DE, or DM and their WISC-IV verbal comprehension score.

Results were similar for the associations that included only prenatal and childhood exposures and corresponding creatinine concentrations (see Supplemental Material, Table S4). Spline regression findings were consistent with the results in Table 5 and showed no contribution by the nonlinear components of the associations (see Supplemental Material, Figure S2). None of the log-scale DAP concentrations in linear models were statistically significant (for the prenatal DE concentration, β = 0.33; 95% CI: –0.12, 0.79, *p* = 0.15; Table 5).

Additional adjustment for birth weight did not modify the associations between the children’s WISC-IV scores and their mothers’ or their own urinary concentrations of DAP, DM, or DE (data not shown). When models were restricted to participants with complete data on all covariates, outcomes, and exposures (*n* = 216) results were generally consistent with findings from the default models for exposures at both time points and associations with working memory and verbal WISC-IV scores (see Supplemental Material, Tables S5 and S6, respectively).

## Discussion

The aim of this study was to assess the potential neurotoxic impact of developmental exposure to OP on cognitive functions in a general population of European children. Neither the mothers’ nor children’s urinary concentrations of either total DAP metabolites or DM metabolites were associated with the children’s WISC-IV scores. Although no association was found between prenatal DE urinary concentrations and the WISC-IV working memory score, our results unexpectedly indicated higher WISC-IV verbal comprehension scores at age 6 years in association with the highest maternal DE urinary concentrations. The 6-year DE urinary concentrations were associated with lower scores of WISC-IV working memory, but not with the verbal comprehension score.

In the Center for the Health Assessment of Mothers and Children of Salinas (CHAMACOS) cohort study, the mean concentration of total DAP metabolites measured in maternal urine samples (taken around 13 and 26 weeks of pregnancy), modeled as continuous (log10 scale) or categorical (in quintiles), was related to lower scores in all WISC-IV subdomains at 7 years of age (Bouchard et al. 2011). They observed significantly lower WISC-IV scores once the second quintile of maternal total DAP concentrations (55 nmol/L) (Bouchard et al. 2011), which is close to the median DAP value measured in our population (43.9 nmol/L). Prenatal DM and DE urinary concentrations in our cohort were both approximately half those measured in the CHAMACOS cohort (see Supplemental Material, Table S7). However, the association between total DAP urinary concentrations and WISC-IV scores in the CHAMACOS cohort was weaker when based on maternal urine samples collected around 13 weeks of pregnancy (range, 5–27 weeks), which is more consistent with the time of maternal urine sample collection for the present analysis (< 19 weeks of gestation).

In the Mount Sinai Hospital (New York) birth cohort, third-trimester pregnancy DE urinary concentrations were associated with lower WISC-IV perceptual reasoning scores among children 6–9 years old (–3.51; 95% CI: –7.31, 0.30 with log_10_-concentration, *n* = 140) and lower working memory scores among children 7–9 years old (–3.48; 95% CI: –7.29, 0.34 with log_10_-concentration, *n* = 114). Prenatal DM urinary concentrations were significantly associated with lower perceptual reasoning scores in the subgroup of maternal *PON1* Q192R QQ genotype participants (–6.15; 95% CI: –11.99, –0.31 with log_10_-concentration, *n* = 39; Engel et al. 2011). As in our study, the authors did not observe significantly lower WISC-IV verbal comprehension scores. The prenatal DM and DE urinary concentrations measured in our cohort are slightly lower than those reported in the Mount Sinai cohort (see Supplemental Material, Table S7). We did not assess perceptual reasoning.

A third U.S. cohort, which enrolled inner-city mothers from New York, observed significantly poorer scores for WISC-IV working memory and a lower total IQ score at 7 years of age associated with higher umbilical cord concentrations of chlorpyrifos, which degrades into DE metabolites (Rauh et al. 2011).

These three American birth cohorts assessing the association of child cognitive functions and OP developmental exposure were all conducted among ethnic minorities or populations with low socioeconomic status. These populations might have been exposed to other factors that enhance their vulnerability to neurotoxic insults and that are not easily adjusted for in association studies. In comparison, our population was composed mainly of well-educated women, only 6% of whom reported they were single. In 2003, according to a national perinatal survey, 42.6% of the pregnant women in France had completed at least high school, compared with 84.4% in our study population (Blondel et al. 2006). Despite this enrollment bias, this study has confirmed well-established factors related to child neuropsychological outcomes, including enriching and cognitively stimulating environments, as assessed by maternal education level, mothers’ IQ, HOME score, or child elementary school (for working memory domain), as well as birth weight, which may be a marker of individual nutritional and social status (Caldwell and Bradley 1984; Dauncey and Bicknell 1999; Weiss et al. 2006). Moreover, enrollment in the PELAGIE birth cohort took place all over Brittany and did not represent one particular vulnerable area. Finally, we cannot rule out the possibility that the stimulating environments and the possible healthier lifestyles in our study population might attenuate the potential neurotoxic insults of OP. Nor, however, can we rule out the possibility that manifestations of OP neurotoxicity may be temporarily masked by compensatory processes in our study population and will become more apparent later in life. Finally, in addition to possible lower exposure levels, another explanation might be potentially distinct exposure pathways or exposure mixtures, as suggested by the higher relative contribution of the DMP metabolite to total DM concentrations in this study, compared with the CHAMACOS cohort (see Supplemental Material, Table S7).

In view of what we know about the potential neurodevelopmental toxicity of OP in children, the positive association between prenatal DE urinary concentrations and the WISC-IV verbal comprehension score must be interpreted with caution. Recently Yolton et al. (2013), who enrolled approximately 400 pregnant women from the Cincinnati, Ohio, area, who were socioeconomically diverse and with similar or slightly higher urinary DAP concentrations (at around 16 and 26 weeks of pregnancy) than in our study (see Supplemental Material, Table S7), found enhanced attention in 5-week-old infants with higher levels of DE exposure. Furthermore, they observed less lethargy and a reduction of autonomic stress with higher DE maternal urinary concentrations. Because DAP metabolites are found in food, the authors suggested that the cognitive benefits of improved socioeconomic status and better nutritional quality may outweigh the potentially detrimental effect of OP developmental exposure.

Our results suggested that DE urinary concentrations measured in 6-year-old children were associated with decreased WISC-IV working memory scores measured at the same age. Given the variability of OP urinary biomarkers, particularly among children (Attfield et al. 2014), this result must be interpreted with caution and requires replication. To our knowledge, only one U.S. birth cohort has assessed children’s cognitive functions in relation to postnatal urinary biomarkers of OP exposure. In the CHAMACOS study, postnatal OP exposure, measured by total DAP metabolites in children’s urinary samples, taken at different ages (i.e., 6 months, 1, 2, 3.5, and 5 years of age), was not associated with WISC-IV scores, except for the 1-year-old DAP urinary concentrations, which were related to better verbal comprehension and IQ scores at 7 years of age (Bouchard et al. 2011).

The limitations of our study are mainly related to OP exposure assessment and the possibility bias due to misclassification or error measurement. OPs are characterized by a short biological half-life. Once absorbed they are rapidly metabolized and eliminated in hours to days. Spot urine samples therefore represent recent OP exposure rather than chronic cumulative exposure. Furthermore, the observation of large intraindividual day-to-day variations of OP urinary biomarkers suggests that repeated urinary measures enable better characterization of OP exposure, especially among children (Attfield et al. 2014; Bradman et al. 2013; Griffith et al. 2011). Moreover, because DAP metabolites are found in food (Lu et al. 2008; Zhang et al. 2008), measurements of urinary levels of DAPs reflect intake of both the toxic parent OP compound and the metabolite itself, without possible distinction. In this sense, fruit and vegetable consumption were thus considered as potential confounders in the present study. Another limitation is the existing imprecision in laboratory measurements. Despite these limitations, urinary biomarkers remain a simple, noninvasive tool for assessing OP exposure; these work best when background exposure levels are relatively stable over time, and have been associated with neurodevelopmental outcomes in previous studies. Future studies should also consider PON1 susceptibility when assessing OP developmental neurotoxicity, in view of the polymorphisms known to be involved in OP detoxification. Because our study involved multiple comparisons in the analysis without correction for them, we cannot exclude the possibility of a chance finding. Finally, simulation studies suggest that the imputation approach we used might induce distortion in variance estimates and thus in conclusions of statistical significance (but with unbiased parameter estimates) when censored values are > 30% (Lubin et al. 2004). This might explain the absence of statistical significance in the association between continuous DE concentrations and WISC-IV scores. This study has many strengths, mostly related to its longitudinal design, allowing us to obtain a substantial quantity of information about prenatal life and childhood and thus to adjust for several major confounders in our analysis. It is the first European study of associations between prenatal biomarkers of OP exposure and child neurodevelopment, assessed by standardized cognitive scales.

## Conclusion

Using first-trimester urinary biomarkers of OP exposure and standardized cognitive tests to assess the potential developmental neurotoxicity of OPs in the European general population, we did not observe associations between biomarkers of prenatal OP exposures and children’s cognitive scores at age 6 years, except that DE exposure appeared to be associated with better verbal comprehension scores. Compared with the previous longitudinal birth cohort studies, our study population is well educated and may thus have fewer risk factors and more compensatory stimulation. Potential distinct exposure pathways, exposure levels, or mixtures are other possible explanations. Although replication is required, our study suggests that postnatal OP exposure may negatively influence working memory scores.

## Supplemental Material

(930 KB) PDF
