# Supplementary material for: Organophosphate Insecticide Metabolites in Prenatal and Childhood Urine Samples and Intelligence Scores at 6 Years of Age: Results from the Mother–Child PELAGIE Cohort (France)
Source: Environ Health Perspect. 2015 Sep 22;124(5):674–80. doi: 10.1289/ehp.1409472 (PMC4858392; doi:10.1289/ehp.1409472)
Supplement: (930 KB) PDF [file ehp.1409472.s001.acco.pdf]

Environ Health Perspect
DOI: 10.1289/ehp.1409472

Note to Readers: *EHP* strives to ensure that all journal content is accessible to all readers. However, some figures and Supplemental Material published in *EHP* articles may not conform to 508 standards due to the complexity of the information being presented. If you need assistance accessing journal content, please contact ehp508@niehs.nih.gov. Our staff will work with you to assess and meet your accessibility needs within 3 working days.

Supplemental Material

Organophosphate Insecticide Metabolites in Prenatal and Childhood Urine Samples and Intelligence Scores at 6 Years of Age: Results from the Mother-Child PELAGIE Cohort (France)

Chloé Cartier, Charline Warembourg, Gaïd Le Maner-Idrissi, Agnès Lacroix, Florence Rouget, Christine Monfort, Gwendolina Limon, Gaël Durand, Dave Saint-Amour, Sylvaine Cordier, and Cécile Chevrier

Table of Contents

Chemical analyses

For the maternal samples (by the LABOCEA Institute, in 2008)

Table: Summary of the analytical method characteristics at three levels (for maternal samples).

For the children's samples (by the LABOCEA Institute, in 2013)

Table: Summary of the analytical method characteristics at three levels (for the children's samples).

Table S1. Descriptive characteristics assessed at inclusion in the cohort of the families participating (n=231) and not participating (n=246) in the 6-year neuropsychological follow-up (PELAGIE cohort, France)

Table S2. Univariate analyses for studying the association between WISC scores of 6-year-old children and covariates (n=231; PELAGIE cohort, France)

Table S3. Associations between organophosphate urinary metabolites and WISC working memory scores (n=231; PELAGIE cohort, France) with minimal adjustment

Table S4. Associations between organophosphate urinary metabolites and WISC verbal comprehension scores (n=231; PELAGIE cohort, France) with minimal adjustment

Table S5. Associations between organophosphate urinary metabolites and WISC working memory scores among participants with complete data (n=216; PELAGIE cohort, France)

Table S6. Associations between organophoshate urinary metabolites and WISC verbal comprehension scores among participants with complete data (n=216; PELAGIE cohort, France)

Table S7. Prenatal urinary organophosphate metabolite concentrations (in nmol/L) across cohorts addressing the potential role of exposure to organophosphate insecticides during pregnancy on neurodevelopment

Figure S1. Spline regressions for studying the associations between organophosphate urinary metabolites and WISC working memory scores (n=231; PELAGIE cohort, France)

Figure S2. Spline regressions for studying the associations between organophosphate urinary metabolites and WISC verbal comprehension scores (n=231 ; PELAGIE cohort, France)

References

Supplemental Material, Chemical analyses

For the maternal samples (by the LABOCEA Institute, in 2008)

For this project, we developed a fully automated method for the determination of urinary metabolites of several pesticides simultaneously (triazines, organosphosphates, carbamates, and chloroacetanilides): automated online sample clean-up by solid-phase extraction/liquid chromatography-electrospray ionization tandem mass spectrometry detection offers cleaner and faster sample preparation and analysis, without either matrix signal suppression or peak broadening.

Reagents and chemicals

Reference standards were purchased from Dr. Ehrenstörfer and from Promochem. LC-MS-grade acetonitrile and methanol were purchased from Fisher (>99%). Analytical grade formic acid was bought from Baker (98%). Nitrogen and argon were purchased from Air Liquide at a minimum purity >99%.

Preparation of standard solutions

The standards solutions of each DAP were prepared in methanol. The internal standards were Di-n-butylphosphate and diuron D6 prepared in methanol. All standards and stock solutions were stored at -20 °C until use. Calibration standards were prepared by adding appropriate working standard solutions to 10 mL fresh sample of DAP-free human urine before extraction to obtain concentrations in the range of calibration.

Sample preparation and extraction procedure

After the urine samples were thawed and shaken, the supernatant was analyzed. The samples (5 mL) were preconcentrated by an automated sample preparation system for high sample volume. The online SPE high volume Symbiosis System (Spark Holland, Netherland) is composed of two units: an automatic cartridge exchange (ACE) module, which hold two trays with up to 96 cartridges, and a high pressure dispenser (HPD) module. The ACE unit is equipped with two clamps and two high-pressure valves. While one cartridge is eluting on the right clamp, the next one is being preconditioned in the left clamp.

The Hysphere C18 HD (2×10 mm) was chosen because it yields the best recovery and retention and the most satisfactory peak shape for the largest number of pesticides. The analytes trapped in the cartridges were eluted with the chromatographic mobile phase.

Chromatographic conditions

Chromatographic separation was performed with a reversed phase Synergi fusion-RP analytical column (250 mm × 2.0 mm, 4 µm particle diameter). The mobile phase was a gradient of a mixture of 5 mM ammonium formiate-0.01% formic acid and acetonitrile 0.01% formic acid. The flow rate was 0.2 mL/min. The chromatographic analysis was performed at 35°C.

Mass spectrometry

LC-MS-MS analyses were performed with a system comprising a Waters alliance 2690 LC pump equipped with an autosampler and connected in series with a Quattro Ultima triple-quadrupole mass

spectrometer from Micromass®, UK. The mass spectrometer was equipped with electropray ionization (ESI). Acquisition was performed in the multiple reaction monitoring (MRM) mode, monitoring two transitions per compound (one for quantification and one for confirmation) in positive ionization mode.

Validation study

All validation procedures were performed with fresh samples of triazine-free human urine. The limit of detection (LOD) was defined as the lowest concentration that the analytical process can reliably differentiate from background levels; it was obtained when the signal was three times the background noise in the chromatograph at the lowest analyte concentration assayed. For the limit of quantification (LOQ), the signal must be ten times the background noise. Based on the characterized ion ratio (one quantitative and one confirmation) of each compound, intra-assay precision and accuracy were assessed at 3 levels in the range LOQ-10 µg/L. If the sample was out this range, it was diluted to be in the range of calibration. In all, five replicate quality control samples of each of the three levels of concentrations were analyzed.

The calibration curves showed good linearity with a correlation coefficient >0.990. The method is precise (CV%≤20%) and accurate. Analytical characteristics resulting from the validation of the method are reported in the table below.

Table: Summary of the analytical method characteristics at three levels (for maternal samples).

Analyte	Accuracy (µg/L)			LOQs		LODs (µg/L)
	Mean±SD					
	Level 1: LOQ µg/L	Level 2: average level µg/L	Level 3: High level µg/L	µg/L	CV% LOQ	
DMP	0.2±0.08	4.4±0.48	11.3±1.13	0.200	17	0.06
DMTP	1±0.4	5.0±0.85	11.5±1.73	1	19	0.32
DMDTP	0.45±0.18	4.20±0.42	11.8±1.18	0.45	20	0.13
DEP	1.25±0.50	4.5±1.04	11.1±2.22	1.25	19	0.366
DETP	1.7±0.68	4.5±0.54	11.6±1.74	1.7	19	0.51
DEDTP	0.02±0.008	4.2±0.42	12.2±1.22	0.02	20	0.01

SD: Standard deviation

For the children's samples (by the LABOCEA Institute, in 2013)

This project used a fully automated method for simultaneous determination of urinary metabolites of pesticides: automated online sample clean-up by solid-phase extraction/Ultra performance liquid chromatography-electrospray ionization tandem mass spectrometry.

Reagents and chemicals

Reference standards were purchased from Dr. Ehrenstörfer and from Cerilliant. LC-MS grade acetonitrile and methanol were purchased from Fisher (>99%). Analytical grade formic acid was

bought from Fisher (99%). Nitrogen and argon were purchased from Air Liquide at a minimum purity >99%.

Preparation of standard solutions

The standard solutions of each DAP were prepared in methanol. The internal standards, Diehylpthiophosphate D10 and dimethylthiophosphate D6, were prepared in methanol. All standards and stock solutions were stored at -20 °C until use. Calibration standards were prepared by adding appropriate working standard solutions to 10 mL fresh sample of DAP-free human urine before extraction to obtain concentrations in the range of calibration.

Sample preparation and extraction procedure

After the urine samples were thawed, shaken, and centrifuged, the supernatant was analyzed. The samples (1 mL) were preconcentrated by an automated sample preparation system. The online SPE is a Waters 2777C sample manager. Waters Oasis HLB Direct Connect cartridge (2.1 × 30 mm) was chosen because it yields the best recovery and retention as well as satisfactory peak shape for these metabolites. The analytes trapped in the cartridges were eluted with the chromatographic mobile phase.

Chromatographic conditions

Chromatographic separation was performed with a reversed phase Waters BEH C18 analytical column (150 mm × 2.1 mm, 1.7 µm particle diameter). The mobile phase was a gradient of a mixture of 0.05% formic acid and acetonitrile 0.05% formic acid. The flow rate was 0.3 mL/min. The chromatographic analysis was performed at 40°C.

Mass spectrometry

LC-MS-MS analyses were performed with a system comprising a Waters Acquity UPLC Binary and a Quaternary pump, connected in series with a Xevo TQ-S triple-quadrupole mass spectrometer from Waters. The mass spectrometer was equipped with electropray ionization (ESI). Acquisition was performed in the multiple reaction monitoring (MRM) mode, monitoring two transitions per compound (one for quantification and one for confirmation) in positive ionization mode.

Validation study

All validation procedures were performed with fresh samples of triazine free human urine. The limit of detection (LOD), defined as the lowest concentration that the analytical process can reliably differentiate from background levels, was obtained when the signal was three times the background noise in the chromatograph at the lowest analyte concentration assayed. For the limit of quantification (LOQ), the signal must be ten times the background noise. Based on the characterized ion ratio (one quantitative and one confirmation) of each compound, the intra-assay precision and accuracy were assessed at 3 levels in the range LOQ-15 µg/L. Samples out of this range were diluted to be in the range of calibration. A quality-control blank (mix of pesticide-free urine) and a quality control sample for each of the three concentration levels in the range of calibration were included every 10 samples. The calibration curve at the LOQ level was verified every 20 samples.

The calibration curves showed good linearity with a correlation coefficient >0.997. The method is precise (CV%≤20%) and accurate. Analytical characteristics resulting from the validation of the method are reported in the table below.

Table: Summary of the analytical method characteristics at three levels (for the children's samples).

Analyte	Accuracy (µg/L) Mean±SD			LOQs		LODs (µg/L)
	Level 1: LOQ µg/L	Level 2: average level µg/L	Level 3: High level µg/L	µg/L	CV% LOQ	
DMP	0.2±0.08	1.07±0.38	9.16±5.49	0.2	16	0.060
DMTP	0.6±0.08	1.67±0.58	17.16±2.6	0.6	13	0.32
DMDTP	0.3±0.11	1.47±0.81	11.72±0.29	0.3	17	0.13
DEP	0.3±0.12	1.44±0.57	15.69±2.93	0.3	20	0.2
DETP	0.3±0.09	1.45±0.43	16.25±5.15	0.3	17	0.1
DEDTP	0.02±0.007	0.09±0.046	11.72±3.63	0.02	18	0.01

SD: Standard deviation

Table S1. Descriptive characteristics assessed at inclusion in the cohort of the families participating (n=231) and not participating (n=246) in the 6-year neuropsychological follow-up (PELAGIE cohort, France)

**Characteristics**	Participants				Non-participants				
	n[a]	%	Mean ± SD	Range	n[b]	%	Mean ± SD	Range	*p*-value[c]
**Characteristics of the pregnant women at inclusion (<19 weeks)**									
Mothers' age	231		30.3 ± 4.1	21.9-44	246		30 ± 4.8	18-44.4	0.46
Maternal educational level									
High school or less	74	32.0			61	46.1			<0.01
University level	157	68.0			184	53.9			
Smoking (% yes)	231	23.4			243	22.2			0.77
Alcohol use (% yes)[d]	231	13.0			241	14.5			0.63
Fish intake (% ≥ 2 per week)	231	29.4			246	22.8			0.10
Fruit and vegetable intake (% ≥ 3 per day)	231	24.2			246	9.8			<0.01
Urinary creatinine levels (mg/L)	231		1080 ± 509	235-3511	225		1111 ± 458	185-3073	0.49
Season of urine collection									0.66
Spring	72	31.2			75	30.6			
Summer	58	25.1			59	24.1			
Autumn	48	20.8			62	25.3			
Winter	53	22.9			49	20.0			
Parity									
0	98	42.4			100	40.8			0.72
≥1	133	57.6			145	59.2			
**Children's characteristics**									
Sex (% male)	231	50.6			246	52.4			0.70
Birth weight (g)	231		3401 ± 436	2340-4660	246		3428 ± 446	2110-4760	0.51

SD: Standard deviation; weeks: weeks of gestation

[a] eligible subjects participating in the 6-year neuropsychological follow-up

[b] eligible subjects not participating in this study because they could not be reached (i.e., lost to follow-up, n=104), refused to participate in the neuropsychological follow-up (n=115), the child had already undergone neuropsychological testing (n=15), or no maternal urine sample was available (n=12)

[c] test (t-test for continuous variables, Chi-square test for categorical variables) comparing the participating and non-participating populations.

[d] Drank an alcoholic beverage at least once a week during pregnancy

Table S2. Univariate analyses for studying the association between WISC scores of 6-year-old children and covariates (n=231; PELAGIE cohort, France).

Characteristics		WISC WMI		WISC VCI	
		Mean or Pearson's Rho	*p*	Mean or Pearson's Rho	*p*
**Characteristics of mothers and families**					
**At inclusion during pregnancy (<19 weeks)**					
Mothers' age	231	0.08	0.21	0.09	0.18
Maternal educational level					
High school or less	74	103.3	0.003	99.2	<0.001
University level	157	109.2		110.7	
Smoking					
No	177	108.3	0.05	107.9	0.11
Yes	54	104.1		104.0	
Alcohol use[a]					
No	201	107.5	0.53	106.8	0.66
Yes	30	105.8		108.2	
Fish intake					
none or <2 per week	163	106.6	0.25	105.2	0.006
≥ 2 per week	68	109.0		111.5	
Fruit and vegetable intake					
< 3 per day	175	106.3	0.05	105.9	0.05
≥ 3 per day	56	110.5		110.7	
Parity					
0	98	106.7	0.57	107.0	0.97
≥1	133	107.8		107.1	
**At the 6-year follow-up**					
Marital status					
No	218	107.1	0.32	107.1	0.43
Yes	13	111.1		107.0	
Mothers' IQ[b]	231	0.27	<0.001	0.39	<0.001
HOME score	231	0.17	0.009	0.20	0.002
Number of children in the family at the 6-year follow-up	231	0.05	0.41	0.02	0.78
**Children's characteristics**					
Sex					
Boy	117	106.2	0.21	107.3	0.80
Girl	114	108.5		106.8	
Birth weight (g)	231	0.09	0.15	0.18	0.006
Breastfeeding duration					
No breastfeeding	80	105.3	0.11	103.1	<0.001
≤ 16 weeks	70	106.8		104.8	
> 16 weeks	81	109.9		112.9	
School at 6 years of age					
Preschool	167	105.4	<0.001	106.9	0.85
Elementary	64	112.4		107.3	
**Testing characteristics**					
Child psychologist					
Psychologist 1	116	110.3	<0.001	107.5	0.67
Psychologist 2	115	104.2		106.6	
Disturbance during test					
No	212	107.7	0.15	107.8	0.01
Yes	19	102.7		98.1	

Weeks: weeks of gestation

p: *p*-value of the Pearson's correlation test for continuous variables or of the statistical test for the simultaneous nullity of coefficients in an univariate variance analysis

[a]Drank an alcoholic beverage at least once a week during pregnancy

[b]Measured with the Wechsler Adult Intelligence Scale

Table S3. Associations between organophosphate urinary metabolites and WISC working memory scores (n=231; PELAGIE cohort, France) with minimal adjustment

Organophosphate metabolites (nmol/L)	n	β (95% CI)
**Pregnancy urinary samples**		
DAP		
< 22.2	77	Ref
22.2-68.8	77	-0.7 (-5.2, 3.9)
> 68.8	77	0 (-4.6, 4.6)
DM		
< 15.5	77	Ref
15.5-59.9	77	-1.6 (-6.1, 3.0)
> 59.9	77	-1.9 (-6.4, 2.7)
DE		
< LOQ	116	Ref
> LOQ -13.2	58	-1.2 (-5.7, 3.3)
> 13.2	57	3.7 (-0.8, 8.2)
**6-year urinary samples**		
DAP		
< 3.95	77	Ref
3.95-25	76	0.9 (-3.7, 5.5)
> 25	78	-1.2 (-5.8, 3.4)
DM		
< LOD	91	Ref
> LOD-13	70	-1.7 (-6.2, 2.8)
> 13	70	-1.4 (-5.9, 3.1)
DE		
< LOD	109	Ref
> LOD -11.1	61	-1 (-5.4, 3.5)
> 11.1	61	-2.6 (-7.1, 1.9)

WISC: Wechsler Intelligence Scale for Children; DAP: dialkyphosphate; DM: dimethylphosphate; DE: diethylphosphate.

Urinary concentrations during pregnancy and during childhood were included simultaneously in the models. The nonlinear component contribution was not tested in these models. The coefficients from linear models of the log-transformed exposures were thus not reported. All models were adjusted for creatinine levels of mother and child.

Table S4. Associations between organophosphate urinary metabolites and WISC verbal comprehension scores (n=231; PELAGIE cohort, France) with minimal adjustment

Organophosphate metabolites (nmol/L)	n	β (95% CI)
**Pregnancy urinary samples**		
DAP		
< 22.2	77	Ref
22.2-68.8	77	1.9 (-3.3, 7.0)
> 68.8	77	1.9 (-3.2, 7.1)
DM		
< 15.5	77	Ref
15.5-59.9	77	-1.3 (-6.4, 3.9)
> 59.9	77	-2.1 (-7.2, 3.0)
DE		
< LOQ	116	Ref
> LOQ -13.2	58	-0.6 (-5.7, 4.4)
> 13.2	57	7.1 (2.0, 12.2)
**6-year urinary samples**		
DAP		
< 3.95	77	Ref
3.95-25	76	2.3 (-2.9, 7.5)
> 25	78	-0.4 (-5.6, 4.7)
DM		
< LOD	91	Ref
> LOD-13	70	-1.4 (-6.5, 3.6)
> 13	70	-3.5 (-8.5, 1.6)
DE		
< LOD	109	Ref
> LOD -11.1	61	-1.2 (-6.2, 3.8)
> 11.1	61	-0.5 (-5.6, 4.5)

WISC: Wechsler Intelligence Scale for Children; DAP: dialkyphosphate; DM: dimethylphosphate; DE: diethylphosphate.

Urinary concentrations during pregnancy and during childhood were included simultaneously in the models. The nonlinear component contribution was not tested in these models. The coefficients from linear models of the log-transformed exposures were thus not reported. All models were adjusted for creatinine levels of mother and child.

Table S5. Associations between organophosphate urinary metabolites and WISC working memory scores among participants with complete data (n=216; PELAGIE cohort, France)

Organophosphate metabolites (nmol/L)	n	β (95% CI)
**Pregnancy urinary samples**		
DAP[a]		
< 22.2	77	Ref
22.2-68.8	77	-0.8 (-5.3, 3.8)
> 68.8	77	-0.4 (-5.0, 4.2)
DM[b]		
< 15.5	77	Ref
15.5-59.9	77	-0.8 (-5.3, 3.8)
> 59.9	77	0.7 (-5.5, 4.1)
DE[c]		
< LOQ	116	Ref
> LOQ -13.2	58	-1.2 (-5.6, 3.2)
> 13.2	57	1.7 (-2.8, 6.2)
**6-year urinary samples**		
DAP[a]		
< 3.95	77	Ref
3.95-25	76	-1.1 (-5.7, 3.5)
> 25	78	-3.4 (-7.9, 1.2)
DM[b]		
< LOD	91	Ref
> LOD-13	70	-0.2 (-4.7, 4.3)
> 13	70	-0.2 (-4.7, 4.3)
DE[c]		
< LOD	109	Ref
> LOD -11.1	61	-2.8 (-7.2, 1.6)
> 11.1	61	-4.8 (-9.1, -0.5)

WISC: Wechsler Intelligence Scale for Children; DAP: dialkyphosphate; DM: dimethylphosphate; DE: diethylphosphate.

Urinary concentrations during pregnancy and during childhood were included simultaneously in the models. The nonlinear component contribution was not tested in these models. The coefficients from linear models of the log-transformed exposures were thus not reported. All models were adjusted for HOME score, breastfeeding duration, mothers' IQ, school, maternal education level, psychologist testing the child, creatinine levels of mother and child, parity, and season of urine collection.

[a]DAP models also adjusted for: maternal alcohol use at inclusion, and disturbances during testing

[b]DM models also adjusted for: maternal alcohol use at inclusion, disturbances during testing, marital status, maternal fruit and vegetable consumption, maternal fish intake, and child's sex.

[c]DE models also adjusted for: marital status, maternal fish intake, and child's sex.

Table S6. Associations between organophoshate urinary metabolites and WISC verbal comprehension scores among participants with complete data (n=216; PELAGIE cohort, France)

Organophosphate metabolites (nmol/L)	n	β (95% CI)
**Pregnancy urinary samples**		
DAP[a]		
< 22.2	77	Ref
22.2-68.8	77	4.1 (-0.8, 9.0)
> 68.8	77	2.1 (-2.9, 7.1)
DM[b]		
< 15.5	77	Ref
15.5-59.9	77	0.4 (-4.6, 5.5)
> 59.9	77	0 (-5.2, 5.3)
DE[c]		
< LOQ	116	Ref
> LOQ -13.2	58	-1.4 (-6.3, 3.4)
> 13.2	57	4.9 (-0.1, 9.8)
**6-year urinary samples**		
DAP[a]		
< 3.95	77	Ref
3.95-25	76	0.7 (-4.3, 5.7)
> 25	78	-2.6 (-7.5, 2.3)
DM[b]		
< LOD	91	Ref
> LOD-13	70	-0.7 (-5.7, 4.2)
> 13	70	-3.5 (-8.4, 1.4)
DE[c]		
< LOD	109	Ref
> LOD -11.1	61	-1.4 (-6.3, 3.5)
> 11.1	61	-2.2 (-7.0, 2.5)

WISC: Wechsler Intelligence Scale for Children; DAP: dialkyphosphate; DM: dimethylphosphate; DE: diethylphosphate.

Urinary concentrations during pregnancy and during childhood were included simultaneously in the models. The nonlinear component contribution was not tested in these models. The coefficients from linear models of the log-transformed exposures were thus not reported. All models were adjusted for HOME score, breastfeeding duration, mothers' IQ, school, maternal education level, psychologist testing the child, creatinine levels of mother and child.

[a]DAP models also adjusted for: disturbances during testing

[b]DM models also adjusted for: disturbances during testing, parity, season of urine collection, maternal fruit and vegetable consumption, and child's sex.

[c]DE models also adjusted for: maternal fish intake.

Table S7. Prenatal urinary organophosphate metabolite concentrations (in nmol/L) across cohorts addressing the potential role of exposure to organophosphate insecticides during pregnancy on neurodevelopment.

OP metabolite	Study	n	% ≥ LOD or LOQ[a]	p25	p50	p75	p90
DAP	PELAGIE cohort (eligible for the 6-year follow-up)	477	87.8%	11.4	37.9	87.5	158.5
	**PELAGIE cohort (participants)**	**231**	**91.3%**	**14.5**	**43.9**	**85.6**	**151.2**
	CHAMACOS cohort[b]	590	88.5%	NA	102.8	277.5	732
	MOUNT SINAI cohort[c]	318	97.3%	31.7	81.3	198.1	NA
	HOME cohort[d]	350	100%	NA	63	NA	NA
DM	PELAGIE cohort (eligible for the 6-year follow-up)	477	86.4%	9.0	30.6	72.3	131.5
	**PELAGIE cohort (participants)**	**231**	**89.6%**	**10.5**	**34.3**	**71.9**	**115.5**
	CHAMACOS cohort	590	80.2%	NA	74.2	232.7	648
	MOUNT SINAI cohort	318	96.4%	16.0	44.8	149.4	NA
	HOME cohort	350	100%	NA	40	NA	NA
DE	PELAGIE cohort (eligible for the 6-year follow-up)	477	48.8%	<LOQ	<LOQ	10.5	32.8
	**PELAGIE cohort (participants)**	**231**	**49.8%**	**<LOQ**	**<LOQ**	**13.2**	**36.2**
	CHAMACOS cohort	590	74.3%	NA	14.1	32.2	70.9
	MOUNT SINAI cohort	318	87.8%	7.8	20.2	54.6	NA
	HOME cohort	350	93%	NA	9	NA	NA
DMP	PELAGIE cohort (eligible for the 6-year follow-up)	477	80.3%	4.3	19.6	55.7	97.2
	**PELAGIE cohort (participants)**	**231**	**83.5%**	**5.3**	**22.0**	**59.8**	**91.1**
	CHAMACOS cohort	590	50.3%	NA	6.7	37.3	105
DMTP	PELAGIE cohort (eligible for the 6-year follow-up)	477	27.7%	<LOQ	<LOQ	8.3	36.4
	**PELAGIE cohort (participants)**	**231**	**26.4%**	**<LOQ**	**<LOQ**	**6.8**	**26.4**
	CHAMACOS cohort	590	65.6%	NA	28.9	119.7	331
DMDTP	PELAGIE cohort (eligible for the 6-year follow-up)	477	20.3%	<LOQ	<LOQ	<LOQ	11.3
	**PELAGIE cohort (participants)**	**231**	**20.8%**	**<LOQ**	**<LOQ**	**<LOQ**	**9.1**
	CHAMACOS cohort	590	48.6%	NA	<LOD	25.9	137
DEP	PELAGIE cohort (eligible for the 6-year follow-up)	477	18.4%	<LOQ	<LOQ	<LOQ	20.5
	**PELAGIE cohort (participants)**	**231**	**19.0%**	**<LOQ**	**<LOQ**	**<LOQ**	**20.0**
	CHAMACOS cohort	590	60.4%	NA	5.3	16.9	46.2
DETP	PELAGIE cohort (eligible for the 6-year follow-up)	477	5.9%	<LOQ	<LOQ	<LOQ	<LOQ
	**PELAGIE cohort (participants)**	**231**	**6.9%**	**<LOQ**	**<LOQ**	**<LOQ**	**<LOQ**
	CHAMACOS cohort	590	49.1%	NA	<LOD	7.6	24.1
DEDTP	PELAGIE cohort (eligible to the 6-year-old follow-up)	477	35.0%	<LOQ	<LOQ	0.4	5.9
	**PELAGIE cohort (participants)**	**231**	**35.1%**	**<LOQ**	**<LOQ**	**0.5**	**7.9**
	CHAMACOS cohort	590	45.6%	NA	<LOD	2	4.8

NA: not available

[a] CHAMACOS, MOUNT SINAI and HOME cohorts used LOD; PELAGIE cohort used LOQ

[b] CHAMACOS cohort (Bradman et al. 2005; the table reports the urinary DAP concentrations measured in the prenatal sample no.1 collected at the beginning of pregnancy, as in the PELAGIE cohort)

[c] MOUNT SINAI cohort is the Mount Sinai Children's Environmental Health Study (Engel et al. 2011)

[d] HOME cohort study (Yolton et al. 2011; Average of the measurements of two urine samples collected at 16 weeks of gestation and 26 weeks of gestation)

Figure S1. Spline regressions for studying the associations between organophosphate urinary metabolites and WISC working memory scores (n=231; PELAGIE cohort, France)

<table>
<tr><th></th><th>Pregnancy urinary samples</th><th>6-year urinary samples</th></tr>
<tr><td>DAP</td><td>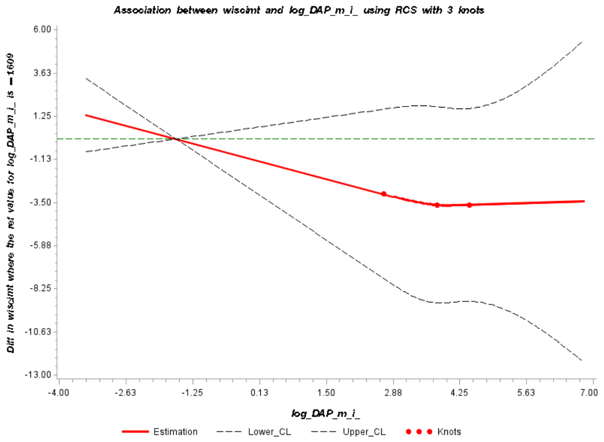

p-value overall association=0.40, non linear component=0.65</td><td>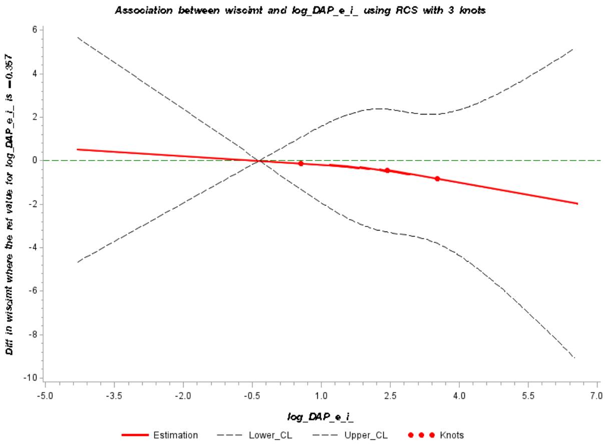

p-value overall association=0.84, non linear component=0.86</td></tr>
<tr><td></td><td colspan="2">Models were adjusted for HOME score, breastfeeding duration, mothers' IQ, school, maternal education level, psychologist testing the child, creatinine levels of mother and child, parity, season of urine collection, maternal alcohol use at inclusion, and disturbances during testing</td></tr>
<tr><td>DM</td><td>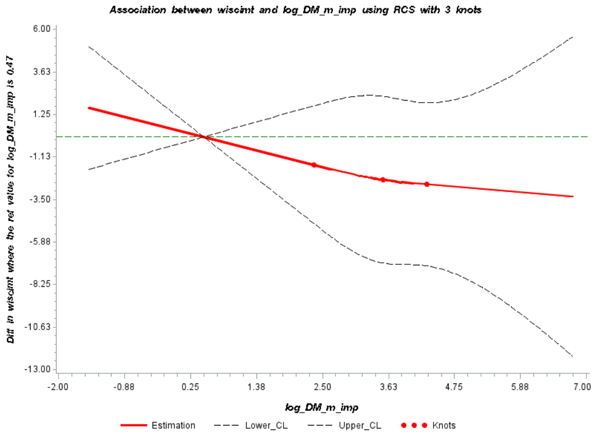

p-value overall association=0.43, non linear component=0.66</td><td>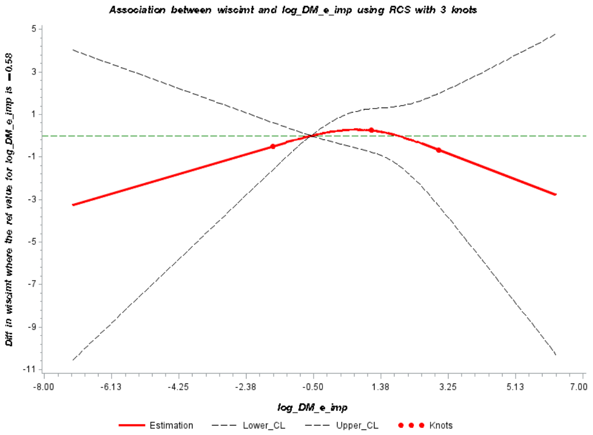

p-value overall association=0.70, non linear component=0.42</td></tr>
<tr><td></td><td colspan="2">Models were adjusted for HOME score, breastfeeding duration, mothers' IQ, school, maternal education level, psychologist testing the child, creatinine levels of mother and child, parity, season of urine collection, maternal alcohol use at inclusion, disturbances during testing, marital status, maternal fruit and vegetable consumption, maternal fish intake, and child's sex.</td></tr>
</table>

DE

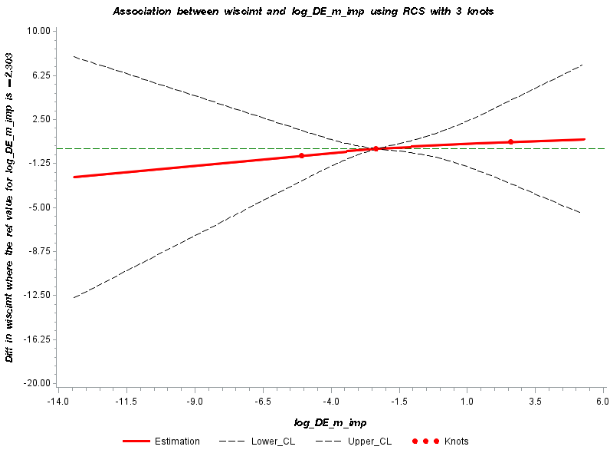

p-value overall association=0.80, non linear component=0.82

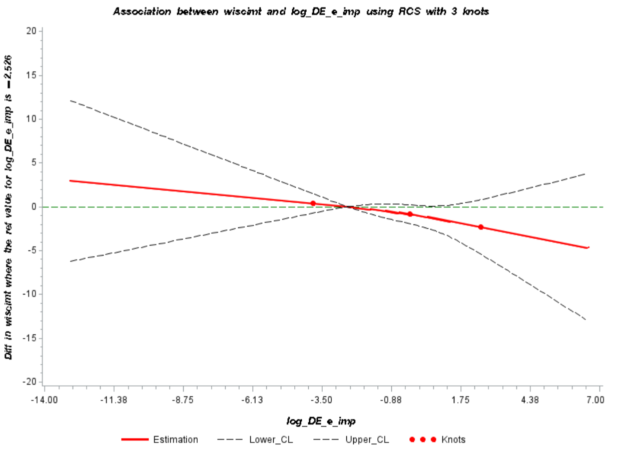

p-value overall association=0.27, non linear component=0.97

Models were adjusted for HOME score, breastfeeding duration, mothers' IQ, school, maternal education level, psychologist testing the child, creatinine levels of mother and child, parity, season of urine collection, marital status, maternal fish intake, and child's sex.

We performed restricted cubic spline regressions, using imputed OP urinary concentrations in natural log-scale with adjustments similar to those obtained in the final regression models provided in the main manuscript. Three knots, located at the 25th, 50th, and 75th percentiles were chosen. The reference value used to calculate the 95% CI was the log of the LOD (or LOQ for maternal urinary concentrations).

Figure S2. Spline regressions for studying the associations between organophosphate urinary metabolites and WISC verbal comprehension scores (n=231 ; PELAGIE cohort, France)

<table>
<tr><th></th><th>Pregnancy urinary samples</th><th>6-year urinary samples</th></tr>
<tr><td>DAP</td><td>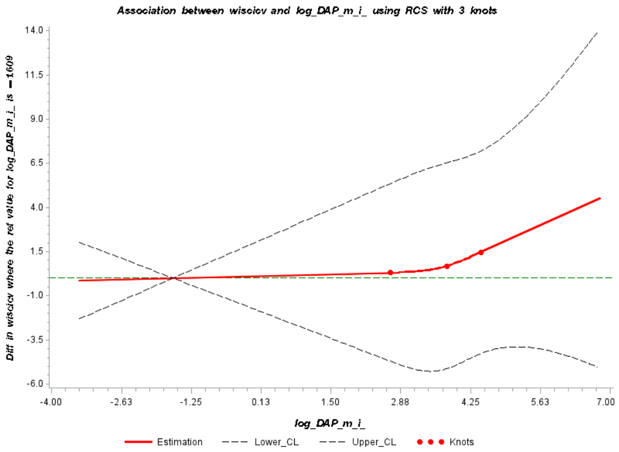

p-value overall association=0.65, non linear component=0.52</td><td>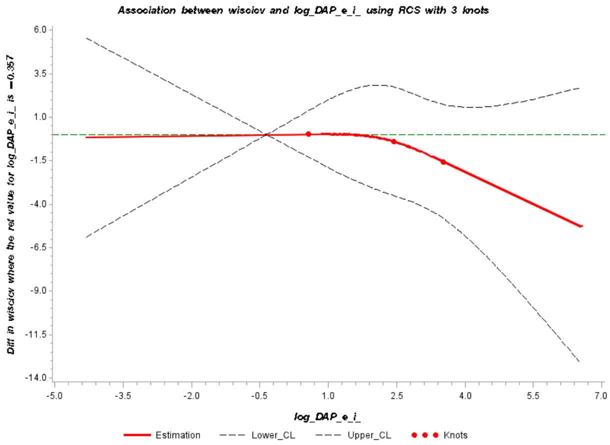

p-value overall association=0.43, non linear component=0.42</td></tr>
<tr><td></td><td colspan="2">Models were adjusted for HOME score, breastfeeding duration, mothers' IQ, school, maternal education level, psychologist testing the child, creatinine levels of mother and child, and disturbances during testing</td></tr>
<tr><td>DM</td><td>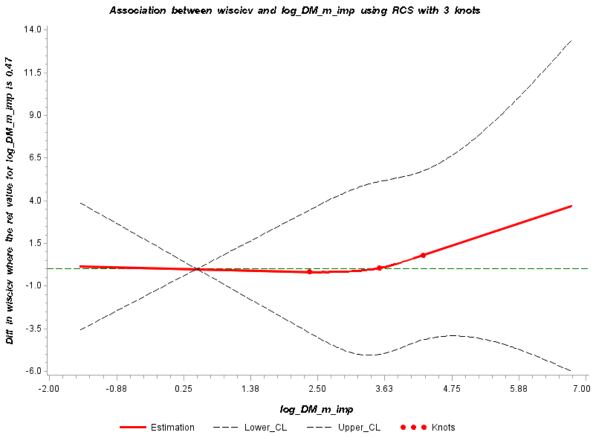

p-value overall association=0.75, non linear component=0.58</td><td>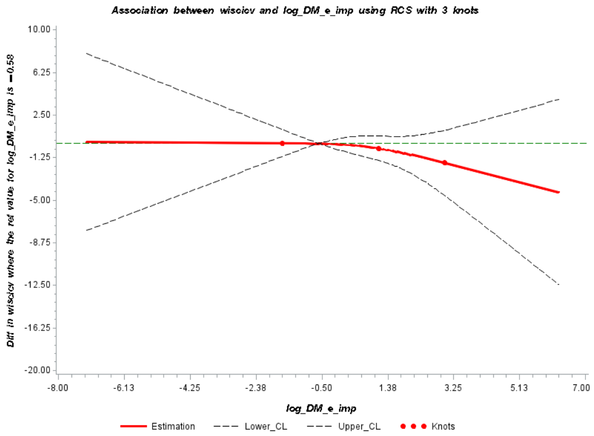

p-value overall association=0.49, non linear component=0.56</td></tr>
<tr><td></td><td colspan="2">Models were adjusted for HOME score, breastfeeding duration, mothers' IQ, school, maternal education level, psychologist testing the child, creatinine levels of mother and child, and disturbances during testing, parity, season of urine collection, maternal fruit and vegetable consumption, and child's sex.</td></tr>
</table>

<table>
<tr>
<td>DE</td>
<td>
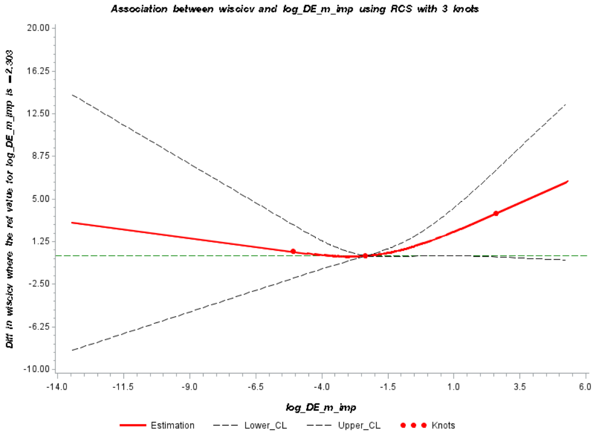

p-value overall association=0.14, non linear component=0.20
</td>
<td>
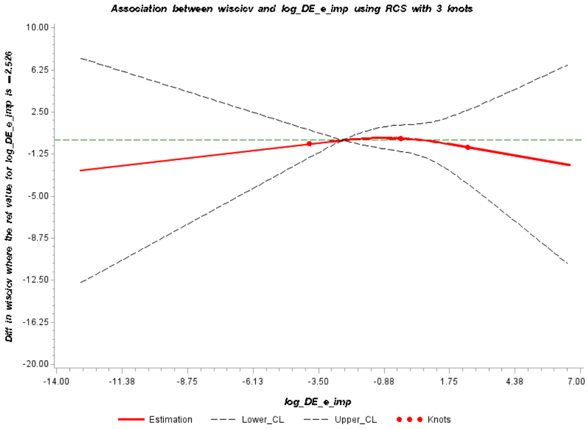

p-value overall association=0.85, non linear component=0.57
</td>
</tr>
<tr>
<td></td>
<td colspan="2">Models were adjusted for HOME score, breastfeeding duration, mothers' IQ, school, maternal education level, psychologist testing the child, creatinine levels of mother and child, and maternal fish intake.</td>
</tr>
</table>

We performed restricted cubic spline regressions, using imputed OP urinary concentrations in log-scale with adjustments similar to those obtained in the final regression models provided in the main manuscript. Three knots, located at the 25th, 50th, and 75th percentiles were chosen. The reference value used to calculate the 95% CI was the log of the LOD (or LOQ for maternal urinary concentrations).

References

Bradman A, Eskenazi B, Barr DB, Bravo R, Castorina R, Chevrier J, et al. 2005. Organophosphate urinary metabolite levels during pregnancy and after delivery in women living in an agricultural community. Environmental health perspectives 113:1802-1807.

Engel SM, Wetmur J, Chen J, Zhu C, Barr DB, Canfield RL, et al. 2011. Prenatal exposure to organophosphates, paraoxonase 1, and cognitive development in childhood. Environmental health perspectives 119:1182-1188.

Yolton K, Xu Y, Sucharew H, Succop P, Altaye M, Popelar A, et al. 2013. Impact of low-level gestational exposure to organophosphate pesticides on neurobehavior in early infancy: A prospective study. Environmental health 12:79.
